# Transcriptional Basis of Copper-Induced Olfactory Impairment in the Sea Lamprey, a Primitive Invasive Fish

**DOI:** 10.1534/g3.118.200920

**Published:** 2019-01-22

**Authors:** Jenna Jones, Kyle Wellband, Barbara Zielinski, Daniel D. Heath

**Affiliations:** * Department of Biological Sciences, University of Windsor, Windsor, Ontario, Canada N9B 3P4; † Great Lakes Institute for Environmental Research, University of Windsor, Windsor, Ontario, Canada N9B 3P4

**Keywords:** copper, olfaction, Petromyzon marinus, brain transcriptome, sea lamprey

## Abstract

Olfaction mediates behaviors necessary for survival and reproduction in fishes. Anthropogenic inputs of contaminants into aquatic environments, specifically copper, are known to disrupt a broad range of olfactory-mediated behaviors and can cause long-lasting damage even at low concentrations that have profound impacts on the biology of aquatic organisms. The sea lamprey (*Petromyzon marinus)* is a primitive fish species invasive to the North American Great Lakes that relies on olfaction to navigate during natal homing and in mate choice during reproduction. To investigate effects of copper on sea lamprey olfaction and the potential for maintenance of olfactory function during copper exposure, we exposed juvenile sea lamprey to environmentally ecologically relevant copper concentrations (0, 5, 10 and 30 µg/L) for 24 hr and characterized gene transcription response in olfactory tissue (*i.e.*, peripheral olfactory organ and olfactory bulb) and forebrain using whole transcriptome sequencing. Copper exposure induced a pattern of positive dose-dependent transcriptional response. Expression changes primarily reflected up-regulation of genes involved in apoptosis and wound healing. Unlike higher vertebrates, genes specifically related to the olfactory senses of the sea lamprey, *e.g.*, olfactory receptors, exhibited little transcriptional response to copper exposure, suggesting the mechanism of copper-induced olfactory impairment is through necrosis of the olfactory bulb and not copper-selective inhibition of olfactory receptors. Fully two-thirds of the differentially expressed genes at higher doses of copper have no known function and thus represent important candidates for further study of the responses to copper-induced olfactory injury. Our results shed light on the evolution of vertebrate olfactory repair mechanisms and have important implications for the conservation and management of both invasive and native populations of lamprey.

Olfaction mediates a variety of behaviors in fishes and plays critical roles in their reproduction, schooling, homing, pheromone detection, and predator avoidance (Tierney *et al.* 2010). In aquatic environments, chemical communication based on olfaction can be disrupted by a variety of environmental pollutants leading to the suppression of these behaviors and a loss of fitness (Atchison *et al.* 1987, Baatrup 1991, Sandahl *et al.* 2007, McIntyre *et al.* 2012). While many studies have examined the consequences (*i.e.*, behavioral effects) that exposure to environmental pollutants have on fish (*e.g.*, Atchison *et al.* 1987, McIntyre *et al.* 2012, Scott and Sloman 2004), few studies have explored the underlying molecular mechanisms that lead to olfactory function disruption (Tierney *et al.* 2010).

Copper has received substantial attention as a contaminant in the aquatic environment due to its highly toxic effects on aquatic organisms (Grosell *et al.* 2003, Vieira *et al.* 2009). It is an active olfactory toxicant at concentrations commonly found in aquatic environments (*i.e.*, levels reported in urban waterways: 2.0 to 64 μg/L; Sandahl *et al.* 2007, Soller *et al.* 2005). Common anthropogenic sources of copper include disc brake pad dust from vehicles, as well as roof and downspout corrosion (He *et al.* 2001). During rain and snow melt events, dissolved copper is washed off roadways and urban landscapes, and enters drainage systems resulting in aquatic habitat contamination (Boulanger and Nikolaidis 2003). Other anthropogenic point sources of copper include industrial effluent and tailings from mining activities. Catastrophic failures at these facilities can result in massive releases of copper pollution into the environment that threaten aquatic life (*e.g.*, the Mount Polley gold-copper mine disaster in British Columbia, Canada; Aug. 4, 2014).

While copper exposure has been shown to disrupt a broad range of fish behaviors and physiology, it specifically targets the olfactory system of fish (Tierney *et al.* 2010). Two mechanisms contribute to the copper-mediated loss of olfactory function as a result of copper exposure. At low concentrations (*e.g.*, <10 μg/L) copper leads to selective toxicity of the olfactory system (Baldwin *et al.* 2003; Sandahl *et al.* 2007; Dew *et al.* 2012, 2014) and induces gene expression changes in the olfactory signal transduction pathway (Tilton *et al.* 2008). At higher concentrations (>25 μg/L) and longer exposures (> 3 hr), olfactory neuron apoptosis and necrosis of olfactory epithelial structures occurs (Wang *et al.* 2013, Hansen *et al.* 1999, Moran *et al.* 1992). The inhibitory effects of copper on olfaction are reversible via the rapid regeneration of neurons, following removal from contaminated water (Moran *et al.* 1992), and some fish appear to regain partial olfactory function despite chronic exposure to low doses (Dew *et al.* 2012). However, the molecular mechanisms that mediate copper toxicity and facilitate recovery from exposure remain poorly characterized.

New molecular genetic technologies such as next-generation sequencing (NGS) are increasingly applied to issues in human health, yet, until recently, few studies have employed these methods to study ecotoxicogenomics in fish (Mehinto *et al.* 2012). With the development of NGS-based gene transcription analyses, environmental stressor effects can be studied at a molecular genetic level across the entire transcriptome for ecologically relevant organisms that often lack a published reference genome. The application of a transcriptomic approach to characterizing the effect of copper on olfaction offers a powerful tool for characterizing the effects of copper exposure.

The sea lamprey (*Petromyzon marinus)* is a primitive species of fish that is native to the northern Atlantic Ocean and the Mediterranean Sea. Throughout its native range, populations of sea lamprey are at risk and conservation efforts have been implemented to address their decline (Pereira *et al.* 2010). In contrast to its conservation status in native areas, the sea lamprey has successfully invaded the Laurentian Great Lakes of North America where invasive populations have caused significant damage to native fish stocks, leading to population declines and loss of economic opportunities for commercial and recreational fisheries (Lawrie 2011). One component of invasive lamprey control efforts in the Great Lakes relies on trapping sea lamprey homing to spawning streams, an olfactory-based behavior (Vrieze *et al.* 2011). Specifically, Great Lakes lamprey abatement strategies include a male-sex pheromone odor application into streams to lure ovulating female into traps or streams ill-suited for offspring survival (Johnson *et al.* 2009). Copper exposure negatively affects the olfactory system (Jones 2016) and this could have an impact on olfactory-based behaviors such as migration and homing. Thus, copper exposure may reduce the success of abatement strategies for lamprey that rely on olfaction.

The sea lamprey also represents an important model for understanding the evolution of olfactory systems. Along with hagfish, lampreys represent the most primitive extant vertebrates. The sea lamprey olfactory system possesses a more diverse array of chemosensory receptors than invertebrates but lacks an entire class of pheromone receptors (V2Rs) that are found in all higher vertebrates (Libants *et al.* 2009). To date, the mechanisms of copper-induced olfactory impairment in aquatic organisms are solely known from teleost fish and whether similar molecular mechanisms underlie copper toxicity in jawless fishes remains unclear. The recent sequencing of the lamprey genome (Smith *et al.* 2013) provides an important resource for transcriptomic studies. The use of a transcriptomic approach will facilitate not only determining mechanisms of copper-induced olfactory impairment for the management of this invasive species, but also will enhance our fundamental understanding of the olfactory system in this primitive vertebrate.

We applied transcriptome sequencing (“RNAseq”) to the olfactory organ of sea lamprey exposed to environmentally relevant levels of copper. This approach provides a comprehensive assessment of the transcriptional response to copper in the olfactory system. We hypothesized that copper will cause two types of response, one involved in adaptive repair or compensation, and another representing non-adaptive damage leading to down-regulation of genes involved in olfactory signaling, coupled with up-regulation of apoptotic processes within olfactory sensory neurons. The current study uses a novel approach to study the effects of copper toxicity at a molecular level in the olfactory system, providing insight into the sea lamprey transcriptomic response to a common aquatic environmental contaminant.

## Methods

### Animal care and maintenance

Sea lampreys (transformer phase) were obtained from United States Geological Survey Hammond Bay Biological Station in Millersburg, Michigan. Animals were transferred to the University of Windsor, and held in the Central Animal Care Facility, according to University of Windsor Animal Care Guidelines (AUPP#14-05). Animals were maintained in aquaria with re-circulating filtration, and water quality was monitored daily. The lampreys were acclimated to these conditions for four months before the experimental tests, copper concentrations during acclimation were measured at below 0.2 µg/L.

### Copper exposure

Copper chloride (CuCl_2_; Sigma-Aldrich Canada Co., Oakville, ON) was diluted into 10L tanks, with target concentrations of 5 µg/L, 10 µg/L, and 30 µg/L. Exposure trials were conducted in these 10 L tanks with recirculated water, while temperature was maintained at 9 **°**C. Water samples were taken after the exposure trial for later copper concentration determination to confirm dose. Groups of four sea lampreys were exposed for 24 hr to the three concentrations of copper. One control group of four lampreys were handled identically; however, not exposed to a copper treatment.

### Collection of olfactory tissue, RNA isolation and sequencing

Immediately following the 24-hour copper exposure, each fish was deeply anesthetized with 150 mg/L MS-222 (Sigma-Aldrich Canada Co., Oakville, ON), and decapitated. Heads were placed under a dissecting microscope, where the nasal cavity was cut to expose the peripheral olfactory organ, olfactory bulb, as well as forebrain. These structures were removed and immediately placed in RNAlater (Thermo Fisher Scientific Inc., Streetsville, ON) and held at 4**°**C for 24 hr, after which they were stored at -80**°**C until total RNA extraction.

Tissue samples were thawed, mechanically homogenized with glass beads in 0.75 mL TRIzol (Thermo Fisher Scientific Inc., Streetsville, ON) and total RNA extraction was carried out following protocols established by Chomezynski and Sacchi (1987). Total RNA integrity was assessed using an Agilent 2100 Bioanalyzer (Agilent Technologies Inc., Santa Clara, CA). RNA samples were also tested for contamination using the absorbance ratio at A260/A280. For each of the 16 olfactory RNA samples, 15 µg of total RNA was aliquoted for library preparation. Total RNA samples were shipped on dry ice to BGI Americas at University of California, Davis (Sacramento, CA, USA) where RNA sequencing libraries were prepared and sequenced using 100 bp pair-end sequencing on an Illumina HiSeq 2000 platform.

### Transcriptome assembly

We used a reference-guided transcriptome assembly approach, implemented in Trinity v3.0.3 (Grabherr *et al.* 2011), to assemble our own transcriptome while leveraging the sequence information present in the existing sea lamprey genome assembly. Briefly, sequences from all samples were pooled and adaptor sequence and sliding-window quality trimming was performed using Trimmomatic v0.32 (Bolger *et al.* 2014) with the default parameters. Pooled and trimmed reads were then mapped onto the lamprey genome assembly (Petromyzon marinus 7.0; NCBI GenBank accession: GCA_000148955.1) using Tophat v2.1.0 (Kim *et al.* 2013). Putative gene transcripts were then assembled from the mapped sequences using the reference-guided approach implemented in Trinity v3.0.3 (Grabherr *et al.* 2011) with a maximum intron size of 20 000 bases. To generate counts of the number of reads per transcript as a measure of gene expression for each sample, sequencing reads for each sample were mapped to the assembled reference transcriptome using Bowtie v1.1.0 (Langmead *et al.* 2009). Effective transcript abundance was then estimated using RSEM and rounded to the nearest integer (Li and Dewey 2011). To avoid issues associated with high variance in read number for genes expressed at low levels, we excluded genes that did not meet a minimum expression threshold of one count per million sequences in at least four samples (one experimental group).

### Whole transcriptome response to copper toxicity

We characterized patterns of gene transcription response for the three copper challenge trials by identifying differentially expressed gene transcripts (relative to control samples) using generalized linear models (GLMs) implemented in the edgeR v3.16.5 package (Robinson *et al.* 2010) in R v3.3.3 (R Core Team 2017). Negative binomial GLMs were fit gene by gene to count data for individuals that had been scaled by a size-factor to control for sequencing library size variation among samples. The GLMs incorporated gene-specific dispersions parameters estimated from the data using an empirical Bayes approach (McCarthy *et al.* 2012). We tested for significant responses between the control and 5 µg/L Cu treatment animals, between the control and 10 µg/L Cu exposure, and finally between the control and 30 µg/L Cu exposure. Significance of the treatment factor was assessed using likelihood ratio tests, and a false discovery rate procedure (FDR = 0.05) was used to correct p-values for multiple comparisons (Benjamini and Hochberg 1995).

### Candidate gene transcription response to copper toxicity

Although whole-transcriptome response to copper exposure in lamprey is important for our understanding of the process, a candidate gene approach also was included to test for dose-response patterns in genes known or suspected to have a role in olfaction and that may be sensitive to copper. Specific olfactory receptor genes have been previously identified in the sea lamprey (Libants *et al.* 2009) and are hypothesized to be a target for copper toxicity where they exhibit a down-regulation of expression in a dose-dependent manner in zebrafish (Tilton *et al.* 2008). We included 23 G-protein coupled receptors (GPCRs) expressed in olfactory sensory neurons including: olfactory receptors (ORs; N = 12), trace amine-associated receptors (TAARs; N = 4), as well as vomeronasal pheromone receptors (V1Rs; N = 7). Individual gene sequences for these receptors were obtained from Libants *et al.* (2009) and were identified in our transcriptome data based on sequence homology using BLAST. We retained all transcripts identified using BLAST with a minimum E-value of 1x10^−5^, indicating significant matches between candidate gene and transcripts in our transcriptomic data.

### Functional annotation and gene ontology enrichment

Functional annotation of analyzed transcripts was accomplished using Blast2GO v3.1 software (Conesa *et al.* 2005). First, sequence similarity searches for each analyzed transcript were performed using the blastx algorithm against the swissprot database and only hits with an E-value smaller than 1x10^−5^ were retained. Gene ontology (GO; Ashburner *et al.* 2000) terms describing gene function that were associated with each unique differentially expressed gene was identified using the default parameters in Blast2GO v3.1.

We tested for GO biological process term enrichment to determine if specific biological functions were over-represented by differentially expressed genes at the different concentrations of copper exposure. To account for length bias in the detection of differentially expressed genes that is present for RNAseq data we tested for GO term enrichment using the goseq v1.26.0 package (Young *et al.* 2010) in R. GO biological process terms obtained from Blast2GO were used to extract all linked ancestral terms in the GO hierarchy for each annotated sea lamprey gene and all of these terms were used to create a custom GO database for all annotated sea lamprey genes. This global set represented the background set against which GO terms associated with differentially expressed genes were tested. A probability weighting function was generated based on the length of each assembled transcript and its status as differentially expressed, or not, for a particular comparison. Statistical significance of over-enrichment of GO terms was then determined using the Wallenius approximation of the null distribution as implemented in the goseq v1.26.0 package. We corrected the resulting p-values with a FDR procedure (Benjamini and Hochberg 1995) and determined statistical significance at FDR = 0.05.

### Data availability

The raw sequencing data are available at the NCBI SRA under the following project accession: PRJNA358429. The lamprey genome is available under the NCBI GenBank accession: GCA_000148955.1. Scripts for conducting the transcriptome assembly, read mapping, and gene expression analyses are available as a GitHub repository: https://github.com/kylewellband/lamprey_rnaseq. Supplemental Tables, Figures, and the expression counts matrix are available on Figshare: https://doi.org/10.25387/g3.7376780.

## Results

### RNA isolation, sequencing, and transcriptome assembly

All 16 total RNA samples were of high quality (RIN ≥ 7.0, 28S- 18S rRNA ≥ ratio: 1.0, (A260:A280 > 1.9). Sequencing generated a range of 15.9 – 24.7 million paired-end reads per sample. Transcriptome assembly using Trinity v3.0.3 software produced an initial total of 286 011 transcripts. Following filtering based on a minimum level of expression of one count per million in one treatment/control group (at least 4 individuals), 29 167 putative genes (transcripts) were analyzed for differential expression.

### Whole transcriptome response to copper toxicity

**Figure 1:**
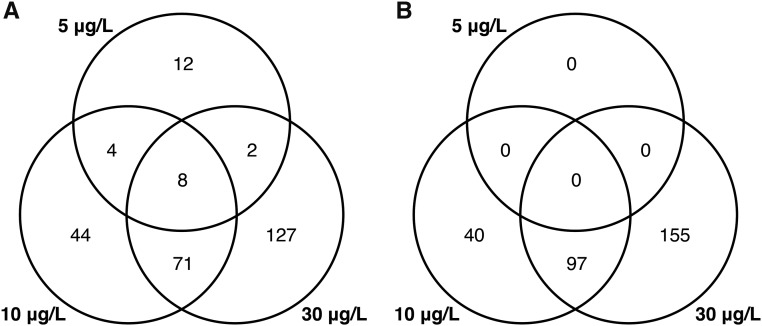
Venn diagrams showing A) the pattern of the differentially transcribed genes (FDR < 0.05) in response to copper exposure shared among the three copper exposure concentrations, and B) the pattern of shared gene ontology biological process in response to three doses of copper exposure.

Transcripts responding to copper exposure were identified as statistically significantly (FDR ≤ 0.05) differentially expressed based on read number for each copper concentration relative to the control samples. As predicted, more genes showed a significant transcriptional response as copper exposure dose increased (Figure 1A). A total of 26 differentially expressed genes were identified at the lowest copper concentration of 5 µg/L, 127 genes were differentially expressed at 10 µg/L, and at the highest dose of Cu exposure (30 µg/L), 208 differentially expressed genes were identified. A considerable number of genes (71) were differentially expressed at both the 10 and 30 µg/L doses of copper and eight genes were significantly differentially expressed at all doses (Figure 1A).

**Figure 2:**
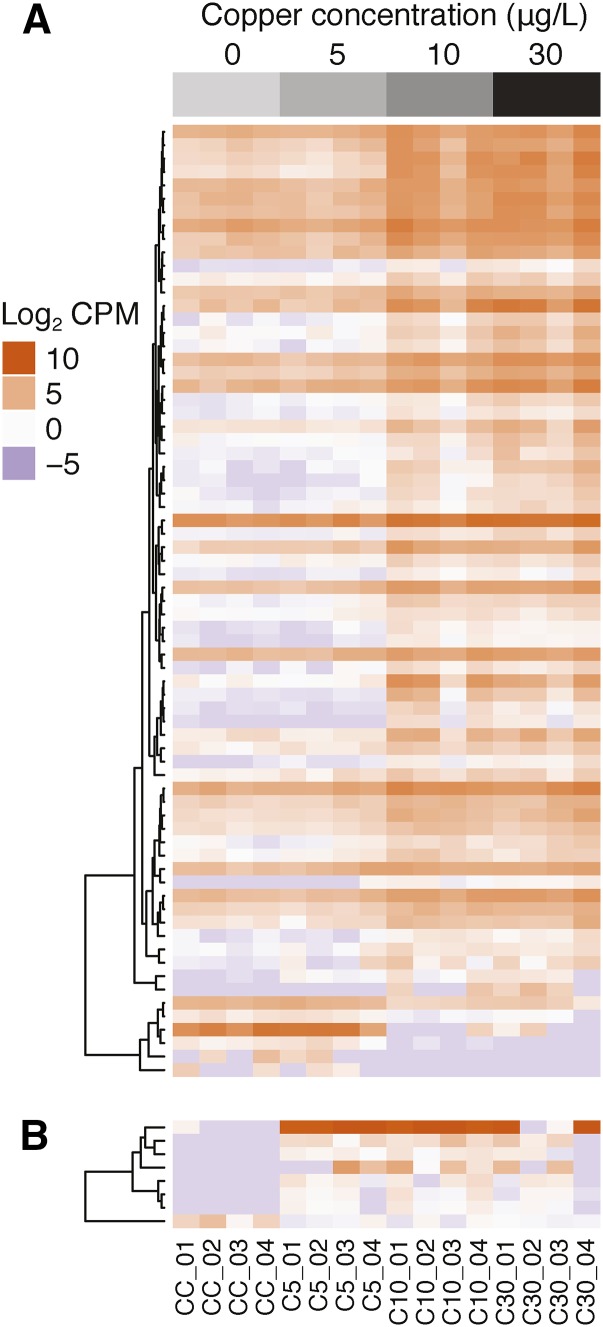
Transcript concentration (Log_2_ counts per million transcripts [CPM]) for (A) 71 genes differential transcribed in the olfactory tissue of lamprey at both 10 and 30 μg/L doses of copper, and (B) 8 genes differentially transcribed in the olfactory tissue of lamprey at all doses of copper.

The primary direction of response was upregulation of gene expression as a result of copper exposure (Figure 2; Figures S1–S3). Only 1/8 genes shared by all treatments and 5/71 genes shared by the 10 and 30 µg/L treatments exhibited downregulation in response to copper exposure. The patterns were more variable for genes only differentially expressed in only one treatment (Figures S1–S3). In particular, a greater proportion of genes (14/26) were downregulated at 5 µg/L compared to greater proportions of genes upregulated at 10 µg/L copper (105/127) and 30 µg/L copper (159/208).

### Candidate gene transcription response to copper toxicity

**Figure 3:**
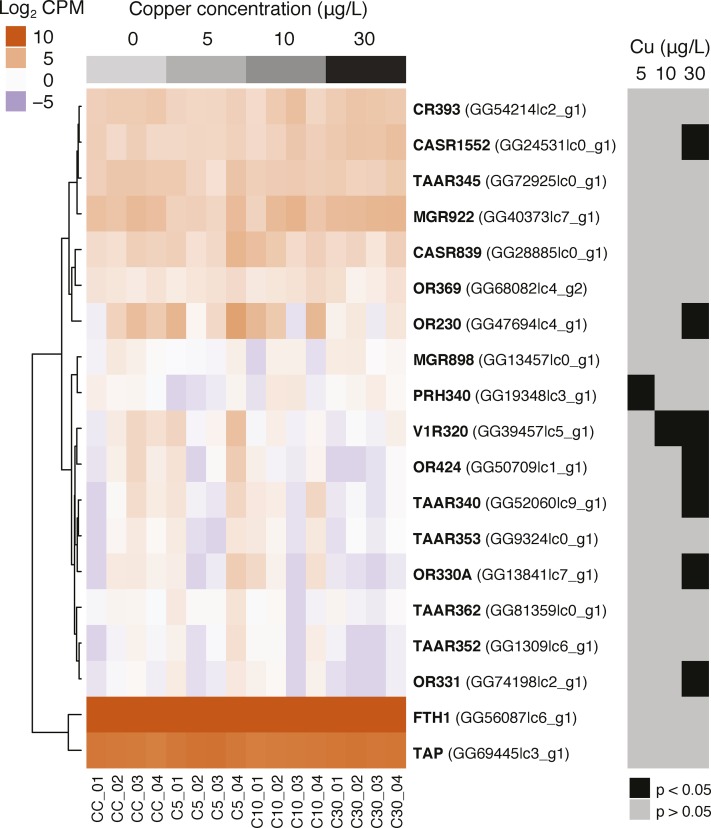
Transcript concentration (Log_2_ counts per million transcripts [CPM]) for 19 candidate genes in the olfactory tissue of lamprey exposed to increasing doses of copper. Statistical significance of differences for each gene x treatment comparison indicated by gray and black boxes to the right.

Sequences for the 23 candidate genes selected from the literature were obtained from the public database for the sea lamprey genome and were identified in our transcriptome data using nucleotide BLAST (e-value cutoff < 1 × 10^−5^). Of the 23 candidate genes we interrogated, 19 were successfully identified in our final sea lamprey transcriptome data allowing us to test for the effect of copper on their transcription levels. Generally, these candidate genes showed low transcriptional sensitivity to exposure to copper (Figure 3). However, seven genes were identified as significantly differentially expressed at the highest copper exposure of 30 µg/L (Figure 3). These differences primarily reflected down-regulation of expression (Figure 3). The down-regulated genes included four olfactory receptors (*ORs 230*, *330A*, *331*, *424*), one trace-amine receptor (*TAAR 340*), and one pheromone odor receptor (*V1R 320*). The sole up-regulated gene was a calcium-sensing receptor (*CASR 1552*).

### Functional annotation and gene ontology enrichment

Of the 29 167 putative gene transcripts that we analyzed, 11 872 were successfully associated with GO annotations. Consistent with the dose-dependent increase in differentially transcribed genes, enrichment testing revealed an increasing number of over-represented gene ontology categories with increasing copper concentration. There was no statistically significant enrichment of gene ontology terms at the lowest dose of copper (5 µg/L); however, there was substantial overlap in the gene ontology terms enriched at 10 and 30 µg/L copper doses (Figure 1B; Table 1). Over-represented terms at these concentrations reflected response to oxidative stress, programmed cell death (apoptosis), and various processes related to wound healing and tissue repair (*e.g.*, immune system responses, angiogenesis, tissue proliferation; see Tables 1, S2, S3). The majority of the differentially expressed genes detected for any dose of copper possessed no annotation information (182/269, Table S1).

**Table 1:** Gene Ontology biological processes over-represented by genes differentially transcribed (FDR < 0.05) at both 10 and 30 µg/L doses of copper exposure (N = 97). To aid in visualization, processes were manually grouped into broad functional categories. GO ID = Gene Ontology process identification number, N_DE_ = Number of differentially expressed genes with GO annotation, N_Total_ = Total number of genes with GO annotation, FDR = false discovery rate corrected significance.

GO ID	Description	N_DE_	N_Total_	FDR
*Growth and Development*				
GO:0008283	cell proliferation	14	2026	0.003
GO:0033002	muscle cell proliferation	6	266	0.006
GO:0048660	regulation of smooth muscle cell proliferation	5	156	0.006
GO:0048659	smooth muscle cell proliferation	5	169	0.006
GO:0045597	positive regulation of cell differentiation	11	1347	0.006
GO:0042127	regulation of cell proliferation	12	1640	0.006
GO:0002040	sprouting angiogenesis	4	90	0.008
GO:1903706	regulation of hemopoiesis	7	509	0.010
GO:0090049	regulation of cell migration involved in sprouting angiogenesis	3	37	0.010
GO:0061061	muscle structure development	10	1245	0.012
GO:0002042	cell migration involved in sprouting angiogenesis	3	40	0.012
GO:0001503	ossification	7	554	0.012
GO:0043542	endothelial cell migration	5	233	0.014
GO:0043534	blood vessel endothelial cell migration	4	125	0.016
GO:0001525	angiogenesis	6	411	0.017
GO:1903670	regulation of sprouting angiogenesis	3	52	0.020
GO:0009888	tissue development	14	2880	0.026
GO:0010631	epithelial cell migration	6	486	0.029
GO:0051094	positive regulation of developmental process	11	1818	0.029
GO:0060541	respiratory system development	6	489	0.031
GO:0090132	epithelium migration	6	495	0.031
GO:1901342	regulation of vasculature development	5	317	0.032
GO:0090130	tissue migration	6	506	0.033
GO:0030334	regulation of cell migration	8	971	0.033
GO:0048514	blood vessel morphogenesis	7	743	0.037
GO:0060537	muscle tissue development	7	755	0.039
GO:0060538	skeletal muscle organ development	5	346	0.040
GO:0043535	regulation of blood vessel endothelial cell migration	3	87	0.048
GO:0007519	skeletal muscle tissue development	4	206	0.048
GO:0008284	positive regulation of cell proliferation	6	567	0.048
*Programmed Cell Death*				
GO:0043067	regulation of programmed cell death	13	1830	0.005
GO:0012501	programmed cell death	14	2245	0.006
GO:0010941	regulation of cell death	13	1975	0.006
GO:0006915	apoptotic process	13	1980	0.006
GO:0008219	cell death	14	2380	0.008
GO:2000377	regulation of reactive oxygen species metabolic process	5	203	0.010
GO:1901701	cellular response to oxygen-containing compound	10	1196	0.010
GO:1903426	regulation of reactive oxygen species biosynthetic process	4	103	0.010
GO:0060548	negative regulation of cell death	10	1208	0.010
GO:1901700	response to oxygen-containing compound	12	1852	0.012
GO:1903409	reactive oxygen species biosynthetic process	4	126	0.016
GO:0042981	regulation of apoptotic process	11	1658	0.019
GO:1901214	regulation of neuron death	6	430	0.020
GO:0033554	cellular response to stress	13	2415	0.022
GO:0072593	reactive oxygen species metabolic process	5	279	0.023
GO:0010942	positive regulation of cell death	8	886	0.024
GO:0070997	neuron death	6	486	0.029
GO:0043523	regulation of neuron apoptotic process	5	316	0.032
GO:2000379	positive regulation of reactive oxygen species metabolic process	3	75	0.035
GO:0051402	neuron apoptotic process	5	347	0.040
*Immune response*				
GO:1902107	positive regulation of leukocyte differentiation	6	165	0.001
GO:1903708	positive regulation of hemopoiesis	7	267	0.001
GO:0002763	positive regulation of myeloid leukocyte differentiation	4	59	0.004
GO:0002682	regulation of immune system process	12	1536	0.005
GO:1902105	regulation of leukocyte differentiation	6	323	0.008
GO:0070555	response to interleukin-1	4	98	0.010
GO:0045321	leukocyte activation	8	718	0.010
GO:1903037	regulation of leukocyte cell-cell adhesion	5	252	0.017
GO:0001817	regulation of cytokine production	7	628	0.020
GO:0007159	leukocyte cell-cell adhesion	5	283	0.023
GO:0045639	positive regulation of myeloid cell differentiation	4	160	0.026
GO:0002761	regulation of myeloid leukocyte differentiation	4	159	0.026
GO:0050865	regulation of cell activation	6	472	0.026
GO:0002684	positive regulation of immune system process	8	910	0.026
GO:0001816	cytokine production	7	711	0.032
GO:0045670	regulation of osteoclast differentiation	3	77	0.038
GO:0050727	regulation of inflammatory response	4	192	0.041
GO:0032602	chemokine production	3	83	0.044
GO:0051249	regulation of lymphocyte activation	5	365	0.046
GO:0039694	viral RNA genome replication	2	18	0.047
GO:0006954	inflammatory response	5	364	0.048
GO:0002521	leukocyte differentiation	6	584	0.050
*Response to stimulus*				
GO:1901652	response to peptide	8	551	0.005
GO:0071495	cellular response to endogenous stimulus	12	1613	0.006
GO:0070887	cellular response to chemical stimulus	16	3037	0.006
GO:0009719	response to endogenous stimulus	13	1976	0.006
GO:0009725	response to hormone	10	1191	0.010
GO:0071310	cellular response to organic substance	14	2490	0.010
GO:0010243	response to organonitrogen compound	9	1082	0.019
GO:0046683	response to organophosphorus	4	139	0.020
GO:0010033	response to organic substance	15	3161	0.020
GO:0071417	cellular response to organonitrogen compound	7	660	0.025
GO:0014074	response to purine-containing compound	4	153	0.025
GO:0033993	response to lipid	8	931	0.028
GO:0014070	response to organic cyclic compound	8	952	0.031
GO:1901698	response to nitrogen compound	9	1261	0.037
GO:1901699	cellular response to nitrogen compound	7	804	0.049
*Metabolic processes*				
GO:0010604	positive regulation of macromolecule metabolic process	18	2968	0.001
GO:0051173	positive regulation of nitrogen compound metabolic process	17	2818	0.001
GO:0009893	positive regulation of metabolic process	18	3427	0.002
GO:0031325	positive regulation of cellular metabolic process	17	3137	0.003
GO:0051247	positive regulation of protein metabolic process	13	1705	0.003
GO:0060255	regulation of macromolecule metabolic process	19	4886	0.012
GO:0031323	regulation of cellular metabolic process	19	5114	0.019
GO:0080090	regulation of primary metabolic process	18	4723	0.026
GO:0046890	regulation of lipid biosynthetic process	4	179	0.036
GO:0019216	regulation of lipid metabolic process	5	341	0.040
GO:0019222	regulation of metabolic process	19	5543	0.040
GO:0032270	positive regulation of cellular protein metabolic process	10	1605	0.040
GO:0051246	regulation of protein metabolic process	13	2754	0.049
*Other*				
GO:0048511	rhythmic process	8	395	0.001
GO:0001775	cell activation	9	956	0.010
GO:1903524	positive regulation of blood circulation	4	117	0.014
GO:0007623	circadian rhythm	5	250	0.017
GO:2000142	regulation of DNA-templated transcription, initiation	3	64	0.026
GO:0042330	taxis	8	998	0.038
GO:0045933	positive regulation of muscle contraction	3	75	0.038
GO:0051240	positive regulation of multicellular organismal process	11	1988	0.048
GO:0050806	positive regulation of synaptic transmission	4	211	0.049
GO:0042327	positive regulation of phosphorylation	8	1064	0.049

## Discussion

To investigate the molecular mechanisms of copper-induced olfactory impairment in a primitive vertebrate, we characterized transcriptome-wide patterns of gene expression in sea lamprey olfactory tissue following copper exposure. Olfactory impairment has been shown to result from two different mechanisms: 1) selective inhibition of olfactory signaling pathways mediated by down-regulation of gene expression and, 2) through tissue necrosis of olfactory epithelium leading to loss of olfactory function. Our transcriptomic analyses demonstrated increasing copper concentrations drive a dose-dependent up-regulation of gene transcription for genes related to response to cell death, tissue damage and wound healing, a pattern consistent with tissue necrosis resulting from copper exposure. In contrast, genes specific to the olfactory system exhibited little transcriptional change except at the highest copper dose. Our results suggest the mechanism of copper toxicity in lamprey is likely a result of tissue damage and not copper selective inhibition of olfactory signaling pathways.

We observed that differentially expressed genes in response to copper exposure were enriched for biological functions related to neuronal apoptosis and cell death, as well as wound healing, cell differentiation, and development. These patterns indicate lamprey olfactory tissue is both initiating programmed death of olfactory sensory neurons, while simultaneously preparing to regenerate and replace these tissues. Tissue necrosis and apoptosis of olfactory neurons have been reported in olfactory tissue of teleost fish exposed to copper under concentrations similar to those we used (Moran *et al.* 1992, Hansen *et al.* 1999, Wang *et al.* 2013). These effects are reversible and previous studies have identified rapid proliferation of olfactory sensory neurons following copper exposure, suggesting an adaptive recovery mechanism in the olfactory system following exposure (Moran *et al.* 1992, Julliard *et al.* 1996, Kolmakov *et al.* 2009). Furthermore, there is evidence that teleost fish can recover partial olfactory function despite chronic exposure to copper contamination (Kolmakov *et al.* 2009, Dew *et al.* 2012). This pattern of cell death following copper exposure, with subsequent rapid regeneration of olfactory tissue, has been suggested as a unique adaptation for protecting the brains of fishes from substances absorbed in naked neurons such as olfactory receptors (Moran *et al.* 1992). Our results indicate similar processes occur in lamprey.

The transcriptional patterns of regeneration and development we report are similar to those reported for zebrafish under copper exposures (Tilton *et al.* 2008). However, while their study reported over-representation of transcriptional processes underlying cell differentiation and development, they reported extensive down-regulation of expression of olfactory and signal transduction pathways that we do not observe in our data. It has been previously shown in teleost fish that copper exposure disrupts olfactory responses by blocking voltage-gated ion channels and G-protein coupled receptors (Sandahl *et al.* 2006). In zebrafish, copper exposure causes a coordinated down-regulation of gene expression in the olfactory signal transduction pathway (*i.e.*, olfactory receptors, g-proteins, ion channels) that has been proposed as a mechanism to explain copper toxicity (Tilton *et al.* 2008). While it is not clear that this represents an adaptive response to copper contamination, it is likely less energetically costly to down-regulate expression of olfactory receptor genes than it is to regenerate olfactory epithelium. We observed weak down-regulation of certain olfactory receptors but only at the highest dose of copper. These effects could reflect an adaptive response in lamprey, but it is more likely they reflect a pathological response due to the loss of olfactory sensor neurons caused by tissue necrosis observed at this dose (Jones 2016).

Copper exposure has highly variable and specific effects on olfactory receptors and signaling pathways. For example, contaminant-specific transcriptional responses to copper *vs.* pesticide treatments have been reported in zebrafish olfactory tissue (Tilton *et al.* 2011) and copper has differing strengths of olfactory inhibition for amine *vs.* bile-acid olfactory stimulants in a variety of fish species (Baldwin *et al.* 2003; Sandahl *et al.* 2007; Dew *et al.* 2012, 2014). The absence of lamprey olfactory receptor transcription down-regulation at low copper concentrations could be 1) a limitation of its more primitive olfactory system, 2) that it is a tough, resilient species that does not need a nuanced response, or 3) that it evolved an, as yet, uncharacterized mechanism to cope with copper and other heavy metal contamination. Sea lamprey are known to possess a less derived olfactory system that lacks an entire class of pheromone receptors (V2Rs) compared with teleosts and higher vertebrates (Libants *et al.* 2009). It is conceivable that the mechanisms facilitating selective down-regulating of olfactory receptors in response to copper exposure for jawed vertebrates only evolved following the split from jawless vertebrates. While the specific mechanisms that allow teleosts to detect copper and modulate olfactory receptor expression remain unknown, lamprey may prove to be a useful comparative model to better understand the evolution of such response mechanisms.

More than two thirds of the differentially expressed genes we detected have no known function. It is surprising that the majority of genes that exhibited the most dramatic transcriptional response to copper have not been characterized at a functional level, as considerable effort has been expended in characterizing toxicologically relevant genes. The majority of these uncharacterized genes exhibited up-regulated dose response with increasing copper exposure, consistent with these genes having a role in the adaptive repair mechanisms in the olfactory system. Given that adaptive repair or compensatory mechanisms are relatively poorly described in ecotoxicology, our “anonymous” candidate genes merit further study and may represent a unique lamprey specific copper detoxification system.

Pheromone-based lamprey abatement strategies are an important control strategy for invasive populations of lamprey in the Great Lakes (Siefkes 2017). Our results support the observed copper induced olfactory injury that underlies olfactory impairment for this species at environmentally relevant levels of copper (Jones 2016). Our transcriptomic results further suggest that adaptive repair mechanisms are being activated but it remains unclear if these mechanisms can restore olfactory function under chronic copper exposure, such as that reported in other species (Dew *et al.* 2012). Further studies quantifying olfactory response and the molecular mechanisms of repair over longer time-scales are required to resolve if lamprey are also capable of recovering olfactory function under constant exposure. To increase the efficacy of olfaction-based management strategies it will be important to have knowledge of copper concentrations present in targeted waterways prior to the initiation of abatement activities. Additionally, it would be useful to improve regulation of heavy metal pollution entering waterways to enhance conservation and management of native lampreys as well as other aquatic species.

In summary, our findings show that acute exposures to copper have rapid and profound impacts on the olfactory tissue transcriptome that are directly connected with observed olfactory sensory loss in the sea lamprey (Jones 2016). Our molecular examination of olfactory responses following copper exposure indicates the mechanism of copper toxicity in lamprey is likely tissue damage, and not copper-selective inhibition of olfactory signaling pathways. Lamprey appear to exhibit an adaptive tissue damage repair gene transcription profile, and resolution of the effects of this process will have important implications for conservation and management. Finally, our study contributes to our understanding of the interaction between the olfactory system and aquatic environment, and how that interaction has evolved in fishes. In particular, the identification of unknown function genes expressed in the olfactory regions that are clearly sensitive to environmental copper provides a new direction for both ecotoxicologists, but also olfactory physiologists.
